# Preparation and Optical Properties of PVDF-CaFe_2_O_4_ Polymer Nanocomposite Films

**DOI:** 10.3390/polym15092232

**Published:** 2023-05-08

**Authors:** Sultan Alhassan, Majed Alshammari, Khulaif Alshammari, Turki Alotaibi, Alhulw H. Alshammari, Yasir Fawaz, Taha Abdel Mohaymen Taha, Mohamed Henini

**Affiliations:** 1 Physics Department, College of Science, Jouf University, Sakaka P.O. Box 2014, Saudi Arabia; 2 School of Physics and Astronomy, University of Nottingham, Nottingham NG7 2RD, UK

**Keywords:** nanocomposites, PVDF polymer, CaFe_2_O_4_

## Abstract

In this work, a synthesis technique for highly homogeneous PVDF-CaFe_2_O_4_ polymer films direct from solution was developed. The structural characterizations were conducted using XRD, FTIR, and ESEM experimental techniques. The XRD characteristic peaks of CaFe_2_O_4_ nanoparticles revealed a polycrystalline structure. The average crystallite size for CaFe_2_O_4_ was calculated to be 17.0 nm. ESEM micrographs of PVDF nanocomposites containing 0.0, 0.25, 0.75, and 1.0 wt% of CaFe_2_O_4_ showed smooth surface topography. The direct E_dir_ and indirect E_ind_ band gap energies for the PVDF-CaFe_2_O_4_ nanocomposites were decreased with the additions of 0.0–1.0 wt% CaFe_2_O_4_. In addition, the refractive index (*n*_0_) increased from 3.38 to 10.36, and energy gaps (E_g_) decreased from 5.50 to 4.95 eV. The nonlinear refractive index (n_2_) for the PVDF-CaFe_2_O_4_ nanocomposites was improved with the addition of CaFe_2_O_4_ nanoparticles, exceeding those reported in the literature for PVC, PVA, and PMMA nanocomposites. Therefore, the PVDF-CaFe_2_O_4_ nanocomposites are expected to take the lead in optoelectronic applications because of their unusual optical properties.

## 1. Introduction

Recently, polyvinylidene fluoride (PVDF) polymer nanocomposites have been of great importance in the scientific and industrial community [1,2,3]. This comes because of the distinctive electrical, optical, and mechanical properties of these nanocomposites. The polymer integration with nanomaterials of different sizes and shapes causes a noticeable change in their properties. PVDF has the repeated monomer unit CH_2_=CF_2_ and is a semicrystalline polymer with five different forms (a, b, g, 𝛿, and e). The major crystal forms of PVDF involve different chain conformations, each of which possesses a component of a net dipole moment perpendicular to the chain [4,5,6,7]. The crystallized b phase of PVDF is the most important for piezoelectric applications. The *β* phase has all-trans conformation (TTTT’), although successive –CF_2_ groups must be deflected by 7° in opposite directions from the planar zigzag conformation to accommodate the fluorine atoms [8,9,10,11]. The spatial symmetrical disposition of the hydrogen and fluorine atoms in the chain of PVDF gives rise to unique polarity effects that influence the polymer properties. Moreover, the inclusion of ferrite nanomaterials in PVDF improves the crystallization of the b phase [12,13]. Accordingly, the PVDF-nanoferrite polymer nanocomposites possess strong magnetoelectric properties and ferroelectricity [14,15]. In addition, the optical properties of PVDF nanocomposite films are governed by a variety of factors, including the composition of the film, the concentration and size of the nanoparticles dispersed throughout the matrix, and the processing conditions used to fabricate the film. Manipulating these parameters, it is possible to tailor the optical properties of the film to meet the specific needs of a given application. One of the most important optical properties of PVDF nanocomposite films that can be tailored is their transparency. In order to be an effective solar cell material, the PVDF film must be sufficiently transparent to allow sunlight to pass through and interact with the active layers of the cell. This requires the careful selection of the nanoparticles used in the composite, as well as precise control over their concentration and dispersion within the matrix. By adjusting these characteristics, it is feasible to create PVDF nanocomposite films that exhibit high degrees of transparency, which are perfect and suitable for solar cell applications [16]. Another important optical property of PVDF nanocomposite films is their ability to absorb and convert sunlight into electrical energy. Nanoparticles such as quantum dots or carbon nanotubes can be embedded in the PVDF matrix to enhance the film’s energy conversion efficiency [17]. These nanoparticles can absorb light across a wide range of wavelengths, and through a process known as exciton energy transfer, they are able to transfer that energy to the PVDF matrix, where it can be converted into electrical energy. The size and concentration of these nanoparticles can also have a significant impact on the energy conversion efficiency of the PVDF film. For example, increasing the concentration of carbon nanotubes within the PVDF matrix can increase energy conversion efficiency by increasing the number of potential exciton energy transfer pathways [18]. Additionally, using nanoparticles with carefully engineered energy levels can enhance the efficiency of the energy conversion process by reducing the amount of energy lost in the form of heat.

Many applications require tuning the optical properties of PVDF nanocomposite films. Therefore, several studies have reported the effect of nanomaterials on the optical properties of PVDF polymer nanocomposites. PVDF–graphene oxide (GO) polymer films have been synthesized via the solution-casting route [19]. The inclusion of GO produced higher absorbance for the PVDF nanocomposites. Moreover, GO content revealed a variation in the refractive index for PVDF polymer films. Ajay Pal Indolia and M. S. Gaur [20] prepared polymer nanocomposite films of PVDF/ZnO concerning 0.0–9.0 wt%. The values of direct and indirect energy gaps decreased with the increase in ZnO. On the other hand, the refractive index of PVDF/ZnO films enhanced from 1.51 to 1.71. E. G. El-Metwally et al. [21] prepared PVDF/Li_4_Ti_5_O_12_ polymer films with doping ratios of 0.0 to 2.0 wt% via the casting of solutions. As the concentration of Li_4_Ti_5_O_12_ increased, both direct and indirect band gaps of the films decreased. Moreover, the refractive index and optical susceptibility significantly improved. Anshu Mli Gaur and Dinesh Singh Rana [22] reported the effect of MgCl_2_ addition on the optical properties of PVDF polymer films. The content of MgCl_2_ rose from 0.0 to 8.0 wt% and enhanced the optical energy gap (0.36–1.99 eV). The PVDF polymer nanocomposites filled with CoFe_2_O_4_, CuFe_2_O_4_, and Cu/CoFe_2_O_4_ were prepared via a solution-casting technique [23]. The addition of the nanoparticles to the PVDF polymer caused a noticeable rise in the refractive index of the samples. Moreover, the polarizability of the PVDF nanocomposites improved after the addition of CuFe_2_O_4_ nanoparticles. Moreover, a study utilizing silver (Ag) nanoparticles reported that the incorporation of Ag nanoparticles could enhance the refractive index of the PVDF polymer nanocomposites. The introduction of Ag nanoparticles also increased the composite’s light absorption in the visible range of the electromagnetic spectrum. Furthermore, the concentration of the Ag nanoparticles could be tuned to achieve optimal properties for specific applications [24]. Another study utilized titanium dioxide (TiO_2_) nanoparticles, which enabled the composite to selectively absorb ultraviolet (UV) light below 385 nm. The TiO_2_ nanoparticles also increased the refractive index of the composite, enhancing its optical transparency. However, beyond a certain concentration, the uniform dispersion of nanoparticles in the PVDF matrix was affected, leading to a decrease in the composite’s optical transparency [25].

Calcium ferrite (CaFe_2_O_4_) is a member of the ferrite family and a widely used material in optical applications because of its unique properties [26]. This compound has a spinel structure, where the Ca^2+^ ions occupy tetrahedral sites and the Fe^3+^ ions occupy octahedral sites [27]. The important properties of calcium ferrite make it suitable for optical applications. Firstly, calcium ferrite exhibits good chemical stability and high resistance to chemical and thermal degradation. This property makes it an excellent material for use as a substrate in high-temperature coatings for optical devices. It can also be used as a protective coating for optical components that are exposed to harsh environmental conditions [28]. Secondly, calcium ferrite’s unique magnetic properties make it ideal for use as a magneto-optical material. It exhibits high magneto-optical activity, which is the ability of a material to modify the polarization or phase of light in the presence of a magnetic field. This property is used to design magneto-optical devices, such as isolators, circulators, and optical storage media [29]. Thirdly, calcium ferrite has high refractive index values, which makes it useful in designing optical devices where light needs to be refracted or directed [30]. Calcium ferrite has refractive index values that are comparable to those of commonly used optical glasses. This property makes it an ideal material for use as a lens or prism [31]. Additionally, calcium ferrite’s optical properties can be further modified by doping it with different transition metal ions, such as Co^2+^ and Ni^2+^. This modification creates new optical properties and modulates its behavior as a magneto-optic material. For example, doped calcium ferrite can be used to design multi-functional optical devices that exhibit both magneto-optical and electro-optical effects [32]. Furthermore, calcium ferrite (CaFe_2_O_4_) is an antiferromagnet crystallized in an orthorhombic structure with space group Pnma [33,34]. CaFe_2_O_4_ is reported to show p-type semiconducting behavior alongside a band gap of 1.9 eV [35,36]. Therefore, the integration of CaFe_2_O_4_ into polymer nanocomposites is expected to reduce the energy gap and enhance the refractive index as well as nonlinear optical parameters [37]. 

Based on the promising literature reports concerning PVDF nanocomposites, in this work, a synthesis technique for highly homogeneous PVDF-CaFe_2_O_4_ polymer films directly from solution was developed. The structural characterizations were completed on XRD, FTIR, and ESEM experimental techniques. Moreover, a detailed study of linear and nonlinear optical parameters, such as energy gap, refractive index, and optical susceptibility, was investigated at different contents of CaFe_2_O_4_ (0.0–1.0 wt%). The content of CaFe_2_O_4_ (0.0–1.0 wt%) was selected to improve the optical properties of PVDF films at a high degree of homogeneity. 

## 2. Experimental

The present experimental procedure used PVDF and CaFe_2_O_4_ powder materials of AnalaR grade. First, 1.0 g PVDF powder was dissolved in a 1:1 DMF/acetone mixture at 50 °C for 120 min. A clear solution was completed, and then 0.25, 0.75, and 1.0 wt% of CaFe_2_O_4_ nanopowder were added slowly. By separating the nanoparticles, it is possible to achieve unique attributes in polymer composites, including a simultaneous enhancement in both toughness and optical properties, even at very low filler quantities. To better disperse the nanoparticles within the polymer matrix, the mixture was stirred for 60 min and then transferred to an ultrasonic pass for 30 min. This process helps to optimize the uniformity of the nanoparticle distribution throughout the matrix [38,39,40]. The homogeneous PVDF-CaFe_2_O_4_ solutions were poured into glass petri plates. Finally, the glass plates were inserted into an electric oven at 100 °C for 24 h to obtain polymer films.

The prepared polymer films were placed on a glass holder for the XRD measurements. The mass and excitation volume of the samples were always the same. A Shimadzu XRD 7000 diffractometer (Kyoto, Japan) was used to study the crystal structure spectra of PVDF-café_2_O_4_ films. The diffractometer was operated at a Cu_ka_ wavelength of 1.54056 Å, and the scan range was from 2q = 5.0° to 80°. A Shimadzu FTIR spectrometer-Tracer 100 was used to record the ATR spectra. The samples were placed directly on the ATR, and the measurements wavenumber range was 399–2000 cm^−1^. A Thermofisher Quattro environmental scanning electron microscope was used to obtain the ESEM micrographs (ESEMs). The polymer films were placed on carbon tape and coated with a thin layer of gold. Measurements of optical properties were employed extensively in this study to offer information on electronic states near the band gap. Agilent’s Cary 60 UV-Vis spectrophotometer was used to extract the absorption spectra of PVDF-CaFe_2_O_4_ polymer films. The polymer films were subjected to photon energy with a wavelength range of 190–1000 nm. All measurements were conducted at 300 K.

## 3. Results and Discussion

**Figure 1:**
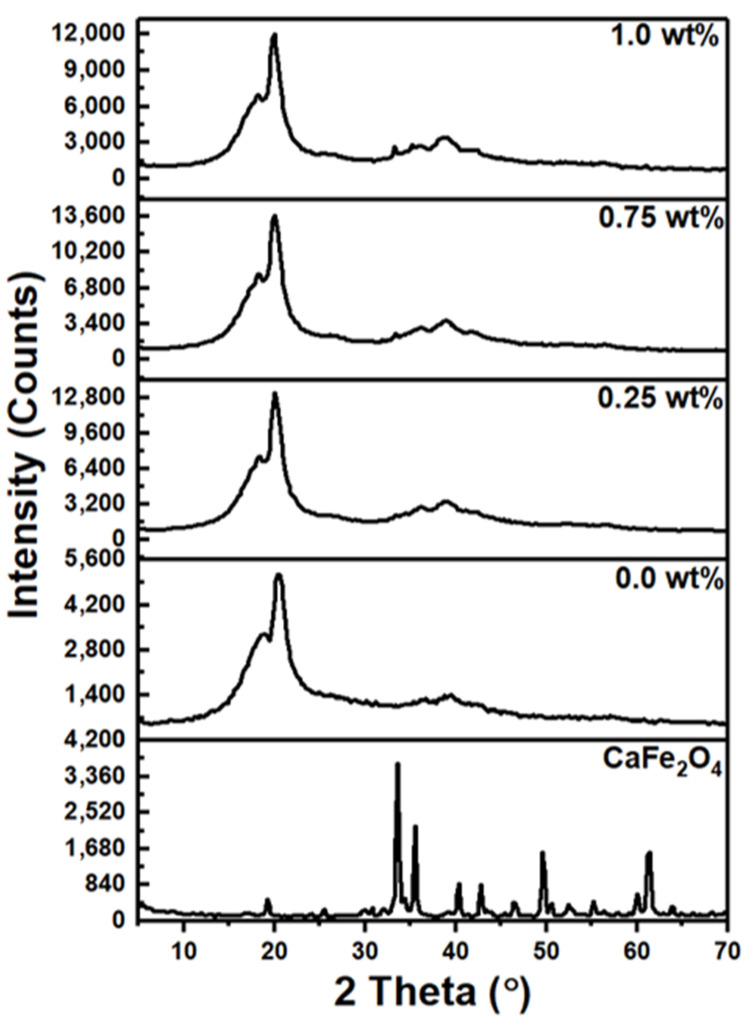
XRD scans for the PVDF-CaFe_2_O_4_ nanocomposite films.

In this work, we utilized the X-ray diffraction patterns of PVDF polymer films–combined CaFe_2_O_4_ nanoparticles at 0.25, 0.75, and 1.0 wt% of CaFe_2_O_4_. Figure 1 shows the XRD patterns for the CaFe_2_O_4_ nanoparticles and PVDF polymer nanocomposite films. The characteristic peaks of CaFe_2_O_4_ nanoparticles reveal a polycrystalline structure. To calculate the crystallite size for CaFe_2_O_4_ nanoparticle, we applied Scherer’s equation concerning the wavelength of X-rays (l = 1.54056 Å), and the peak’s full width at half maximum (*β*) was found to be 17 nm [41,42]:

(1) D=0.9λ/βcosθ 

According to the ICDD card number (65-0678), the crystal structure of the CaFe_2_O_4_ is an orthorhombic phase (Pnam 62 space group), and the cell parameters are a = 9.218, b = 10.679, and c = 3.014 Å. The different crystalline phases of the PVDF-CaFe_2_O_4_ nanocomposite films are observed in an XRD pattern, which belong to the α and *β* phases. The CaFe_2_O_4_ nanoparticles diffraction peaks of the (102), (202), (302), (210), (113), (311), (214), and (512) planes are observed at 2θ = 19.2°, 25.7°, 33.6°, 35.6°, 40.4°, 42.8°, 49.6°, and 61.4°, respectively. The PVDF diffraction peak of the (0 2 0) plane is at 2 theta = 18.6°, which corresponds to the α phase of PVDF. The diffraction peak of the PVDF (200) plane is at 2θ = 20.4°; this plane is the signature of the *β* phase of PVDF. The XRD patterns of the PVDF-CaFe_2_O_4_ blend nanocomposites with 0.25, 0.75, and 1.0 wt% show decreases at 2θ = 18.3°, 18.2°, and 18.1°, respectively, for the α phase with the increasing content of CaFe_2_O_4_. Meanwhile, the intensity of this peak improves as the percentage of CaFe_2_O_4_ increases from 0.0 to 1.0 wt%. Moreover, when the concentrations of the CaFe_2_O_4_ are increasing, the diffraction peak related to the *β* phase of PVDF is detected at 2θ = 20°. The decrease in the peak position illustrates the intercalation at PVDF/CaFe_2_O_4_ interface. Moreover, this shift affects the optical properties of PVDF-CaFe_2_O_4_ polymer films. On the other hand, the diffraction peaks of CaFe_2_O_4_ located at 33.08°, 35.20°, and 38.79° are detected in the XRD spectra of polymer films containing 0.75 and 1.0 wt%. All these outcomes confirm the complexing of the PVDF polymer matrix with CaFe_2_O_4_ nanoparticles. 

**Figure 2:**
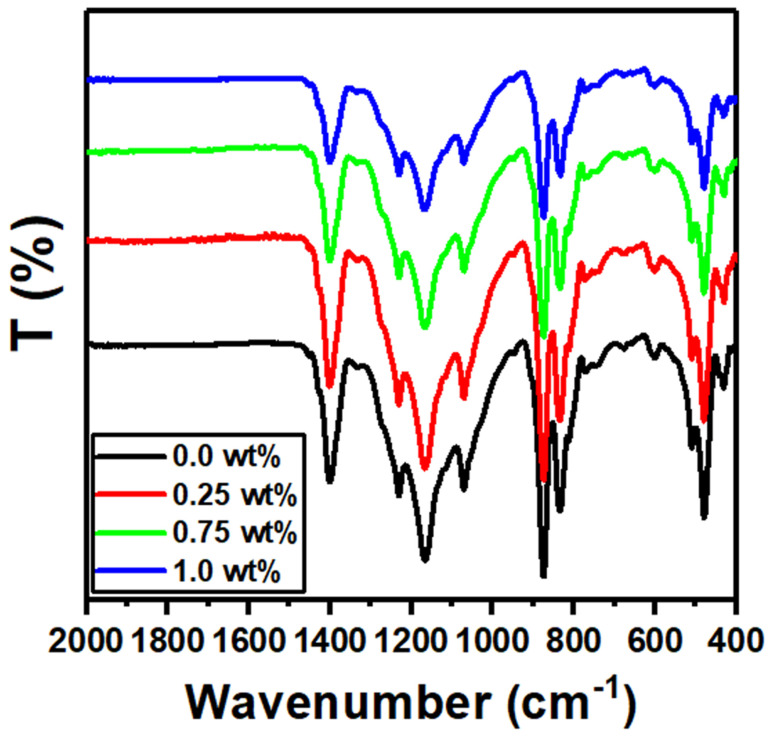
FTIR spectra scans for the PVDF-CaFe_2_O_4_ nanocomposite films.

Figure 2 shows the FTIR spectrum of the PVDF-CaFe_2_O_4_ nanocomposite films in the range of 400 to 2000 cm^−1^. The PVDF-CaFe_2_O_4_ nanocomposite films reveal the presence of α and *β* phases. The α phase of PVDF is observed at 615 cm^−1^ [43]. The distinguished absorption bands at 423, 480, 509, 833, 873, 1070, 1167, 1230, and 1400 cm^−1^ correspond to the *β* phase of PVDF [44]. The increase in CaFe_2_O_4_ content (0.25–1.0 wt%) shows a small shift of peak position toward higher wave numbers for CaFe_2_O_4_ nanocomposite films. The absorption band related to CaFe_2_O_4_ is not detected in the FTIR spectra. Moreover, the positions of all peaks change slightly because of the small content of CaFe_2_O_4_ nanoparticles [45,46]. The data results of XRD and FTIR for the polymer nanocomposite films reveal the interconnection of PVDF and CaFe_2_O_4_.

**Figure 3:**
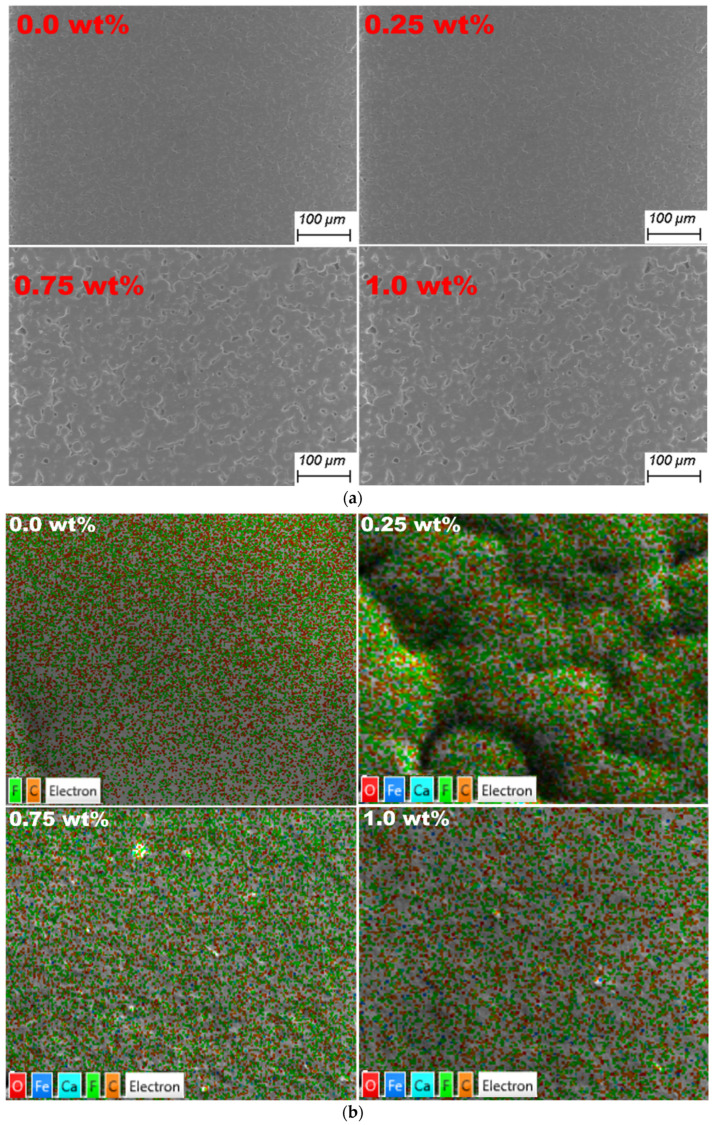
(**a**) The ESEM scans for PVDF-CaFe_2_O_4_ nanocomposite films. (**b**) The EDS mapping scans for PVDF-CaFe_2_O_4_ nanocomposite films.

Figure 3a shows ESEM micrographs of PVDF-CaFe_2_O_4_ nanocomposites containing 0.0, 0.25, 0.75, and 1.0 wt%. The ESEM scans supplied show porous surface topography. Furthermore, the dispersion of CaFe_2_O_4_ dominates the images in Figure 3, as the filler is distributed homogeneously. The EDS mapping data displayed in Figure 3b indicates that the nanocomposite structure is very dense at higher concentrations of CaFe_2_O_4_. This can be explained by the interconnection of nanofiller at the PVDF polymer/CaFe_2_O_4_ interface, as shown in Figure 3b. These outcomes confirm the interaction and complexity of PVDF/CaFe_2_O_4_ nanocomposite polymer structures. 

**Figure 4:**
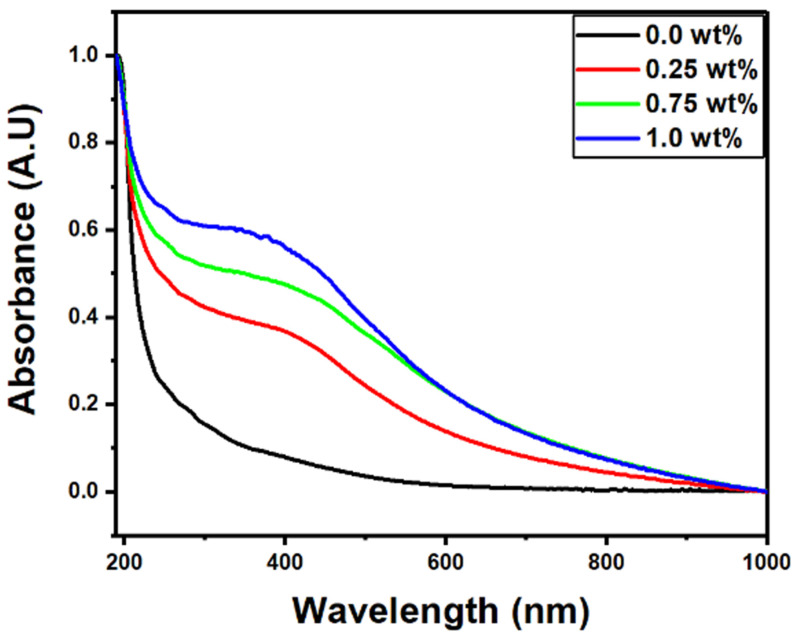
Optical absorption curves vs. wavelength for the PVDF-CaFe_2_O_4_ nanocomposites.

Understanding the optical characteristics of materials is mostly based on their electronic energy band structure. The optical transmission spectrum provides basic information about a material’s optical bandgap. It is one of the most important strategies for selecting an innovative material for optical applications. The PVDF-CaFe_2_O_4_ nanocomposites were subjected to UV–visible spectroscopy to measure their optical properties at 0.0, 0.25, 0.75, and 1 wt% of CaFe_2_O_4_. Figure 4 represents the absorbance as a function of wavelength within the range of 190–1000 nm. Adding more CaFe_2_O_4_ content led to an increase in the optical absorption of the PVDF films. The process of doping polymer with nanoparticles created numerous absorption centers, leading to an overall increase in absorbance.

**Figure 5:**
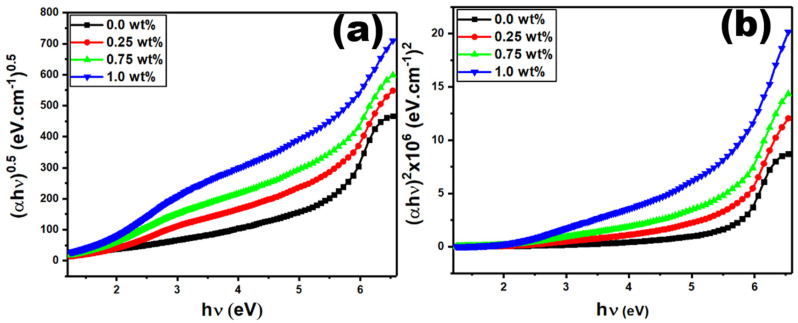
Graphs of (**a**) (αhυ)^2^ vs. hυ and (**b**) (αhυ)^1/2^ vs. hυ for the PVDF-CaFe_2_O_4_ nanocomposites.

When photons are absorbed by a material, the electrons in the valence band can be excited to the conduction band across the band gap. There are two types of optical transitions. The first is a direct transition, in which the electron keeps its momentum, whereas the second is an indirect transition, in which the electron changes momentum by emitting or absorbing a phonon. So far, because indirect gaps need both a phonon and a photon at the same time, their transitions have a substantially lower probability. However, indirect transitions may still exhibit significant absorption magnitude despite having a larger density of states than direct transitions, as the transition frequency is inherently linked to the number of valence and conduction band states. The optical band gaps *E_opt_* are estimated by the Tauc formula as following equation [47,48,49]:

(2) $$\alpha h\upsilon =k{\left(hv-E_{opt}\right)}^{x}$$

where α, hv, and k denote the absorption coefficient, the energy of the incident photons, and a blend structure’s constant, respectively. The letter x denotes both direct and indirect allowed transitions with values of 0.5 and 2, respectively. The direct E_dir_ and indirect E_ind_ band gap energies are determined by extrapolating the linear plot with the x-axis hv values, as shown in Figure 5a,b. Table 1 shows that the direct (E_dir_) and indirect (E_ind_) band gap energies are decreased with the additions of CaFe_2_O_4_ at different concentrations. This reduction in the band gap energy may be due to the decrease in the electron donor nature and polymer backbone conjugation. This also can be explained by the contribution of the states near the band edges, which reduces the Fermi level and affects the energy band gap [22].

**Figure 6:**
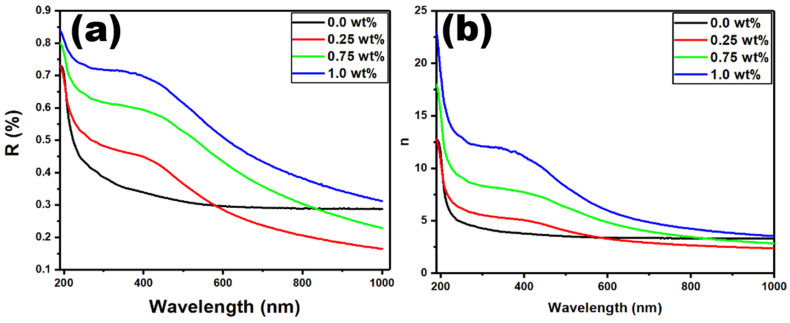
Graphs of (**a**) reflectance and (**b**) refractive index vs. wavelength for the PVDF-CaFe_2_O_4_ nanocomposites.

The optical refractive index is the most straightforward measure for studying optical characteristics. The interaction of light with materials induces optical polarization. The amount to which the electronic structure of the polymer molecules is modified by the optical frequency of the incoming electromagnetic radiation determines the refractive index. Moreover, the refractive index is linked to the electric dipole moment caused by the electromagnetic interaction of component atoms and molecules with light. Figure 6a,b represents the optical reflectance (*R)* and refractive index (*n*) against the wavelength over the range of 200–1000 nm for PVDF-CaFe_2_O_4_ nanocomposites. The reflectance shown in Figure 6a enhances with the increase in nanocrystals; this can be explained by the condensation of CaFe_2_O_4_ inside the polymer network or because of the chemical and physical intermolecular interaction between polymer segments and nanofiller [50]. The values of *n* can be calculated as [51,52]

(3) $$n=\left(\frac{1+R}{1-R}\right)+\sqrt{\frac{4R}{{\left(1-R\right)}^{2}}-k^{2}}$$

The extinction coefficient is represented by k, and it is equal to αλ/4π. Table 1 shows the values of *n*_0_, where they increase with the addition of CaFe_2_O_4_ at different concentrations.

We have used the equation below to demonstrate the refractive index (*n*_0_) [53] (where (hv→0)).

(4) $$n_{0}{}^{2}=\left(1+\frac{E_{d}}{E_{0}}\right)$$

The calculated values of$\;n_{0}$ are represented in Table 1. The estimated $n_{0}$ is improved with the addition of CaFe_2_O_4_ at different contents, where $n_{0}\;$= (3.38 to 10.36). The increase in the refractive index correlates with the increase in the filler density on the surface of the polymer film. These inferred values of the refractive index show variation with changing calcium ferrite concentration and open up a wide range of applications.

It is useful to use refractive index values that change with the energy of the photons to calculate both single-oscillator and dispersion energies. We use the following equation to demonstrate the refractive index dispersion [54,55]:

(5) $${\left(n^{2}-1\right)}^{-1}=\frac{E_{0}}{E_{d}}-\frac{1}{E_{0}E_{d}}\;{\left(h\upsilon \right)}^{2}$$

**Figure 7:**
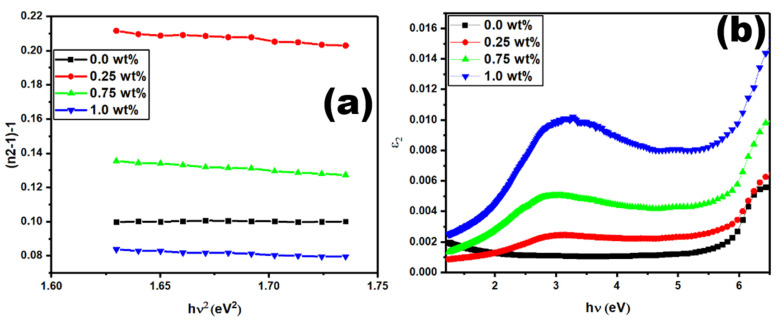
Graphs of (**a**) (n^2^ − 1)^−1^ vs. $h\upsilon^{2}$ and (**b**) Ɛ_2_ vs. hυ for the PVDF-CaFe_2_O_4_ nanocomposites.

The single-oscillator and dispersion energies are represented by *E*_0_ and *E_d_*, respectively. The plots in Figure 7a are used to calculate the values of *E*_0_ and *E_d_,* concerning the slope and intercept of straight lines. Table 1 shows the values of the *E*_0_ and *E_d_*, where they are higher than those of the pure PVDF film. 

The relationship between optical dielectric loss function (ε_2_ = 2nk) and photon energy (hv) is shown in Figure 7b. The intercept of straight lines gives the energy gaps (*E_g_*) of PVDF-CaFe_2_O_4_ nanocomposites that are listed in Table 1. The recommended charge transitions are direct transitions because the values of *E_g_* are close to E_dir_, as shown in Table 1. 

The strength of a single oscillator (*f*) is defined by the relationship below [56,57]:

(6) $$f=E_{d}E_{0}$$

As demonstrated in Table 2, the addition of CaFe_2_O_4_ improves the oscillator strength. A wide range of photonic devices are thought to be provided by the field of nonlinear optics. This stimulates the study of the nonlinear optical parameters of the PVDF-CaFe_2_O_4_ polymer at different nanofiller concentrations. Therefore, the linear optical susceptibility ($\chi^{\left(1\right)}$) and the third order nonlinear optical susceptibility ($\chi^{\left(3\right)}$) of the PVDF-CaFe_2_O_4_ nanocomposites are completed for the nonlinear optical analysis [58,59].

(7) $$\chi^{\left(1\right)}=\frac{E_{d}/E_{0}}{4\pi };\;x^{\left(3\right)}=6.82\times 10^{-15}{\left(E_{d}/E_{0}\right)}^{4}$$

Table 2 lists the values of $x^{\left(1\right)}$ and $x^{\left(3\right)}$, which show a gradual increase with the increasing content of café_2_O_4_ nanoparticles. 

For many optoelectronic applications, the estimation of nonlinear refractive index is very important. Therefore, the nonlinear refractive index (n_2_) for the PVDF-CaFe_2_O_4_ nanocomposites is calculated using $x^{\left(3\right)}$ and n_0_ data based on the following equation [60,61]:

(8) $$n_{2}=\frac{12\pi x^{\left(3\right)}}{n_{0}}$$

The estimated values of n_2_ are given in Table 2, which improves with the addition of CaFe_2_O_4_ nanoparticles. The data acquired for n_2_ are higher than that for PVC [62], PVA [63], and PMMA [64] nanocomposites. Because of their unusual optical properties, PVDF-CaFe_2_O_4_ nanocomposites take the lead in optoelectronic applications.

## 4. Conclusions

In this work, a synthesis technique was developed for highly homogeneous PVDF-CaFe_2_O_4_ polymer films processed directly from solution. Structural characterizations were conducted by various experimental techniques, such as XRD, FTIR, and ESEM. The XRD characteristic peaks of CaFe_2_O_4_ nanoparticles revealed a polycrystalline structure. The crystallite size for CaFe_2_O_4_ was calculated to be 1.54056 Å. ESEM micrographs of PVDF-CaFe_2_O_4_ nanocomposites containing 0.0, 0.25, 0.75, and 1.0 wt% showed smooth surface topography. The E_0_, E_d_, and n_0_ values were enhanced with the addition of CaFe_2_O_4_ to the PVDF. In contrast, the E_dir_, E_ind_, and E_g_ values were observed to decrease as we increased the content of CaFe_2_O_4._ The linear optical susceptibility ($\chi^{\left(1\right)}$) and the third order nonlinear optical susceptibility ($\chi^{\left(3\right)}$) values were increased with the increasing content of CaFe_2_O_4_ into the PVDF. The refractive index of PVDF-CaFe_2_O_4_ nanocomposites exceeds those reported in the literature for PVC, PVA, and PMMA nanocomposites. According to the reported enhancement in the intensity of the *β* phase and unusual optical properties, the PVDF-CaFe_2_O_4_ nanocomposites are highly suggested to take the lead in optoelectronic applications.

## Figures and Tables

**Table 1:** Optical calculations of PVDF-CaFe_2_O_4_ nanocomposites.

CaFe_2_O_4_ Concentrations (wt%)	E_dir_ (eV)	E_ind_ (eV)	E_0_ (eV)	E_d_ (eV)	n_0_	E_g_ (eV)
0.0	5.59	5.2	6.60	68.60	3.38	5.50
0.25	5.48	4.71	7.32	150.13	4.64	5.35
0.75	5.35	4.39	7.69	384.30	7.14	5.10
1.00	5.15	4.18	7.41	787.84	10.36	4.95

**Table 2:** Data of *f*, $x^{\left(1\right)}$, $x^{\left(3\right)}$, and n_2_ for the PVDF-CaFe_2_O_4_ nanocomposites.

CaFe_2_O_4_ Content (wt%)	*f* (eV)^2^	χ^(1)^ (esu)	χ ^(3)^ (esu)	n_2_ (esu)
0.0	452.49	0.83	8 × 10^−11^	8.9 × 10^−10^
0.25	1098.99	1.63	12 × 10^−10^	9.8 × 10^−9^
0.75	2955.14	3.98	425 × 10^−10^	2.25 × 10^−7^
1.0	5840.71	8.46	870 × 10^−9^	3.17 × 10^−6^

## Data Availability

Data available on request from the corresponding author.
